# Mobile Personal Health System for Ambulatory Blood Pressure Monitoring

**DOI:** 10.1155/2013/598196

**Published:** 2013-05-09

**Authors:** Luis J. Mena, Vanessa G. Felix, Rodolfo Ostos, Jesus A. Gonzalez, Armando Cervantes, Armando Ochoa, Carlos Ruiz, Roberto Ramos, Gladys E. Maestre

**Affiliations:** ^1^Department of Computer Engineering, Polytechnic University of Sinaloa, 82199 Mazatlan, SIN, Mexico; ^2^Department of Computer Science, National Institute of Astrophysics, Optics and Electronics, 72840 Puebla, PUE, Mexico; ^3^Computer Engineering, Technological Institute of Morelia, 58120 Morelia, MICH, Mexico; ^4^Institute for Biological Research and Cardiovascular Institute, Faculty of Medicine, University of Zulia, Maracaibo 4002, Venezuela; ^5^Departments of Psychiatry and Neurology and the Gertrude H. Sergievsky Center, Columbia University, New York, NY 10032, USA

## Abstract

The ARVmobile v1.0 is a multiplatform mobile personal health monitor (PHM) application for ambulatory blood pressure (ABP) monitoring that has the potential to aid in the acquisition and analysis of detailed profile of ABP and heart rate (HR), improve the early detection and intervention of hypertension, and detect potential abnormal BP and HR levels for timely medical feedback. The PHM system consisted of ABP sensor to detect BP and HR signals and smartphone as receiver to collect the transmitted digital data and process them to provide immediate personalized information to the user. Android and Blackberry platforms were developed to detect and alert of potential abnormal values, offer friendly graphical user interface for elderly people, and provide feedback to professional healthcare providers via e-mail. ABP data were obtained from twenty-one healthy individuals (>51 years) to test the utility of the PHM application. The ARVmobile v1.0 was able to reliably receive and process the ABP readings from the volunteers. The preliminary results demonstrate that the ARVmobile 1.0 application could be used to perform a detailed profile of ABP and HR in an ordinary daily life environment, bedsides of estimating potential diagnostic thresholds of abnormal BP variability measured as average real variability.

## 1. Introduction

Chronic noncommunicable diseases (NCDs), such as heart disease, stroke, cancer, chronic respiratory conditions, and diabetes, are the leading cause of mortality in the world, accounting for 63% of all deaths [1]. The leading cause of NCD deaths in 2008 was cardiovascular diseases (CVDs), accounting for 17 million deaths, nearly 30% of global mortality [2]. Thirteen percent of global deaths are attributed to hypertension, the leading risk factor for mortality [2]; the prevalence of hypertension in the global adult population was estimated to be 26% in 2000 and was predicted to increase by about 60% by 2025, to a total of 1.5 billion [3].

Technological innovations that improve prevention and control of CVDs are desperately needed. In this sense, recent studies using the average real variability (ARV) index [4] reported a significant association between high reading-to-reading blood pressure (BP) variability (BPV) and cardiovascular events [4–8]. The ARV is a novel index that has proven to be a more accurate method to assess BPV than the commonly used standard deviation (SD) [4–6, 9, 10]. BPV is a multifaceted phenomenon, influenced by the interaction between external emotional stimuli, such as stress and anxiety, and internal cardiovascular mechanisms that can vary from heartbeat to heartbeat. The complexity of BPV makes analysis difficult, and its independent contribution as predictor of cardiovascular outcomes is not yet completely clear [11]. Nevertheless, monitoring of BPV in daily life might provide a means to control hypertension, ultimately, preventing CVDs. 

Currently available ambulatory blood pressure (ABP) monitors are portable, fully automatic devices that can record BP for 24 hours or longer, while subjects go about their normal daily activities [12]. This technique provides a better estimate of risk in an individual subject than traditional clinical methods, because it (i) removes variability among individual observers, (ii) avoids the “white coat” effect (the transient, but variable elevation of BP in a medical environment [13]) and “masked hypertension” (normotensive by clinical measurement, but hypertensive by ambulatory measurement [14]), (iii) includes the inherent variability of systolic and diastolic BP (SBP and DBP) [15], and (iv) provides information on changes in BP, that is, circadian components. A circadian BP profile with a reduced decrease in nighttime BP level (nondipper status) can indicate increased cardiovascular risk [16–18]. However, the wider use of ABP monitoring, although justified, is limited by its availability and cost. Patients referred to use this technology tend to pay approximately $40 to $70 (USD) per test, depending on the volume of tests performed (i.e., minimum cost per test could only be achieved by a high-volume testing center) [19]. Furthermore, there is currently no experimental or commercial ABP device that estimates BPV through the ARV index.

This paper presents a mobile personal health monitor (PHM) application for ABP monitoring, with the goal of improving health care through early diagnosis of abnormal BP and heart rate (HR) levels, better hypertension control, electronic health registry of individuals, and data for clinical prognosis of CVDs. The paper includes an overview of PHM systems; describes the advantages and limitations of such a mobile health initiative; describes our mobile PHM application in detail, including design considerations and graphic user interface (GUI); discusses the specific context, strengths, and limitations of our approach; proposes future improvements.

## 2. Mobile Personal Health Monitor

**Figure 1:**
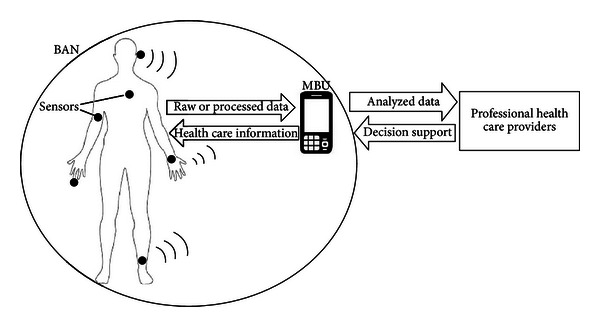
High-level architecture of mobile personal health monitor system.

Mobile PHM systems provide personalized, intelligent, reliable, noninvasive, real-time, and pervasive health monitoring [20, 21]. They are part of a body area network (BAN), integrated by a mobile base unit (MBU), with a set of wearable wireless sensors with on-board processing, wireless data transfer, and energy storage capability (Figure 1). The sensors are attached to the user's body; the collected data may be processed locally within the body sensors and/or remotely via wireless transmission to the MBU. The MBU analyzes the data in real-time and provides immediate feedback and personalized information to the user. The analyzed data can also be sent to professional healthcare providers for medical feedback and to support clinical decisions. Mobile PHM systems offer a set of functions that give them global appeal; however, some features might limit their usability and acceptance.

### 2.1. Pervasive Monitoring

The goal of pervasive monitoring in mobile PHM systems is to provide healthcare services to anyone at any time, overcoming the constraints of place, time, and character [22]. Data processing should be incorporated in the subject's environment in such a way that the interaction between the user and the MBU becomes natural, and the user can obtain personalized information in a totally transparent form.

### 2.2. Integrated Multisensing Platform

Mobile PHM systems can support biosensors that monitor vital signs (e.g., HR, BP, and blood glucose), environmental conditions (temperature, humidity, and light), and location, integrated by a multisensing platform. Light weight and portability allow long-term, unobtrusive, noninvasive, and ambulatory health monitoring.

### 2.3. Real-Time Data Analysis

The mobile PHM software of the MBU stores and analyzes the data received from the sensors in real-time, providing instantaneous feedback to the user. PHM systems could alert the user of abnormal events or abrupt changes in near real time, for example, by vibration, loud sound, and/or flashing screen messages.

### 2.4. Personalized Health Care

Users can configure mobile PHM systems to their specific healthcare needs and preferences, depending on the user's biological profile, as well as the clinical application [23]. Diagnostic thresholds of the risk factor under study could be defined by the user's age, gender, and ethnicity. 

### 2.5. Electronic Data Collection

Mobile PHM systems allow rapid digitization of recorded data, greatly improving quality and efficiency compared to traditional data collection processes with subsequent transcription to computer systems [22]. Continuous monitoring and subsequent transmission to healthcare providers offer an extensive clinical database for data mining analysis of potential risk factors and/or associations among clinical attributes.

### 2.6. Flexible Communication Protocol

The communication protocols of a mobile PHM system are extremely flexible, because local communication within the BAN can use Wi-Fi [24], Bluetooth [25], or ZigBee [26], and remote communication can be performed via 3G [27] or other available internet communication.

### 2.7. Data Security, Confidentiality, and Privacy

Concern about security, confidentiality, and privacy of user health data is an important barrier to the use of mobile PHM systems. Security breaches in data transmission could generate situations where confidential data of a heart patient, for example, are manipulated by malicious attackers; regular readings could be altered to indicate a serious problem, and the resulting inaccurate feedback could even cause the patient to have a heart attack [22]. Alternatively, the data might be of interest to parties not authorized by the user, such as insurance companies or employers, and such access might result in privacy concerns [28]. Thus, policymakers and program managers must be aware of security issues in the mobile health domain, so that appropriate policies and protections can be developed and implemented [29].

### 2.8. Need for Multiplatform Applications

The development environments for mobile phones include various systems, such as Android, BlackBerry, iPhone, and Windows Phone platforms. Each uses a different software language, and applications developed for one environment do not operate in the others [30]. For example, Withings company developed a mobile system that manually registers self-measurements of BP through a wired connection between the sensor and the mobile device, but which only operates in Apple platforms [31]. At present, mobile phones service providers do not compete strongly to offer health-related services. However, expectations of market growth [32–34] could worsen the scenario, because the pursuit of market leadership might further fragment the same. Therefore, multiplatform mobile PHM applications are needed.

### 2.9. Usability and Acceptance among Elderly

While older people are less apt to accept novel and unknown technology than younger people [35], recent studies found that older adults are motivated to use mobile applications if they are satisfactorily informed about the resulting benefits [36, 37]. Elderly people should be the primary target population of mobile PHM for several reasons. First, the global population is aging [38], and chronic NCDs are associated with advanced age [39], so that multimorbidity (coexistence of two or more chronic diseases) is expected to become an increasingly common problem [40]. Second, many elderly people now live alone, with no one to help them to record physical signs like blood pressure [41]. Third, the chances of surviving a fall, heart attack, or stroke are six times greater if the elderly get help within an hour [41]. Finally, mobile PHM systems could provide the elderly with real-time, long-term, nonintrusive assisted living and care services, tailored to their personal health condition [41]. However, acceptance of mobile PHM by older adults is not only based on their health requirements but also on their perspective of technology. Since cognitive performance commonly declines with age, minimizing the complexity of healthcare applications and user-application interactions could be key to the adoption of mobile PHM systems by elderly users and should be considered in their design and development [42]. Therefore, simplicity and motivation seem to be the key factors for usability and acceptance of mobile PHM applications by elderly people.

## 3. A New Mobile PHM for Ambulatory Blood Pressure Variability

### 3.1. Rationale

ARV, a recently tested indicator of reading-to-reading BPV [4], has been shown to be significantly related to cardiovascular outcomes [4–8]. ARV attempts to correct for the limitations of the commonly used SD, which accounts only for the dispersion of values around the mean, and not for the order of the BP readings [4–6, 9, 10]. ARV is particularly useful for examining effects of intermittent stress on the cardiovascular system; intermittent BP load on cardiovascular structures may be as important as tonic BP load [43]. Although for most outcomes, ARV was found to be an independent and better predictor of cardiovascular risk than SD [4–6, 8], no previous study has estimated ARV using real-time monitoring, because up to this point, no commercial or experimental real-time device has been capable of monitoring ABP and determining ARV.

Most current healthcare surveillance technologies and diagnostic tools are used in clinical environments, providing only a snapshot of disease under conditions different from the patient's normal lifestyle. The results could lead to difficult or even incorrect diagnoses [20]. Personalized health monitoring in the patient's own environment could significantly improve early detection of CVDs. Personal ABP monitoring would result not only in more accurate diagnosis hypertension but also more effective monitoring of response to therapy, thereby aiding a more tailored approach to patient monitoring.

**Figure 2:**
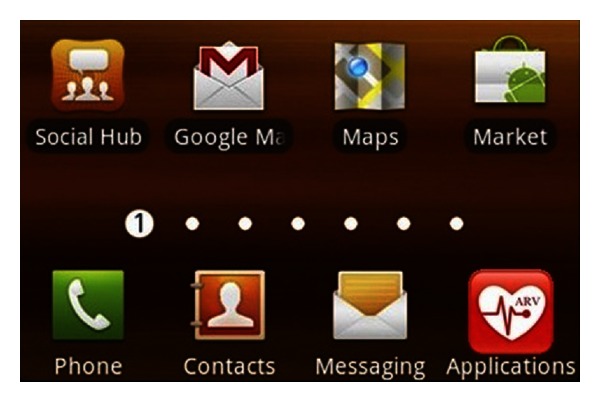
The ARVmobile v1.0. for an Android platform.

The ARVmobile v1.0 is a multiplatform, mobile PHM application for real-time, noninvasive, and long-term ABP monitoring (Figure 2). The ARVmobile 1.0 could (i) provide a detailed picture of ABP and heart rate (HR) in a normal environment, (ii) improve the early detection and intervention of hypertension, (iii) improve treatment of hypertension, (iv) identify unexpected responses to antihypertensive treatment, and (v) provide data for the estimation of diagnostic thresholds of abnormal BPV measured as ARV.

### 3.2. Hardware and Software

**Figure 3:**
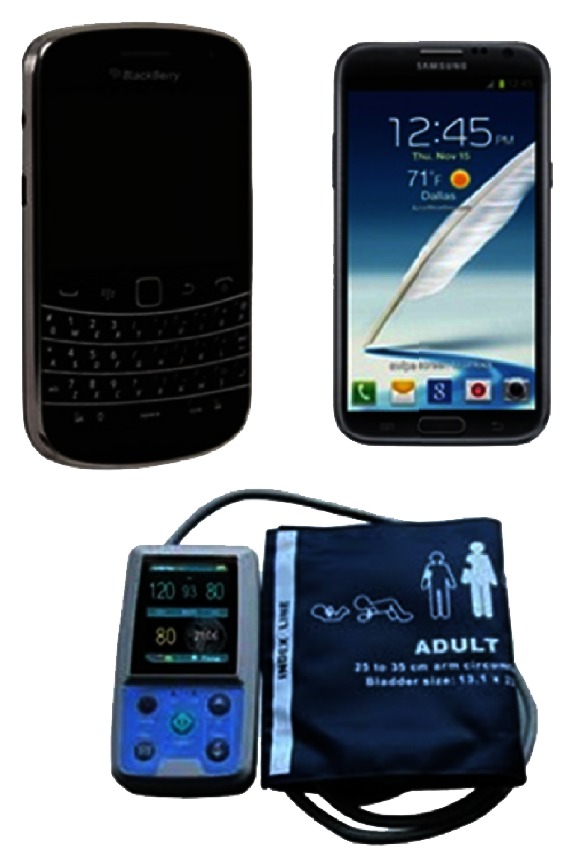
Android and Blackberry smartphones and the ABPM50 device.

The ARVmobile v1.0 was proved in two smartphones (Figure 3). The Samsung I-9100 Galaxy S II, which runs Android OS v2.3 on an ARM Cortex-A9 CPU, with a clock rate of 1.2 GHz Dual Core and 1 GB RAM LPDDR2 [44]. It supports Bluetooth v3.0+HS, Wi-Fi 802.11 a/b/g/n, and USB v2.0; the BlackBerry 9900 Bold that runs BlackBerry OS v7 on a Qualcomm MSM8655 CPU, with a clock rate of 1.2 GHz and 768 MB RAM [45]. It supports Bluetooth 2.1 A2DP/EDR, Wi-Fi 802.11 b/g/n, and USB v2.0. The ARVmobile v1.0 used an ABPM50 [46] to measure BP in millimeters of mercury (mmHg) and HR in beats per minute (bpm). The ABPM50 is an ABP monitor device of low cost that continuously and noninvasively monitors BP level by the oscillometric method [47] and uses Bluetooth for wireless communication. Its sensor has an accuracy of ±3 mmHg and meets ANSI/AAMI SP10-1992 standards [48]. The ABPM50 has a DC power of 3 V (2 AA 1.5V alkali batteries), weighs ~1 kg, and can record more than 600 measurements in 48 hours, in programmable time intervals of 15, 30, 60, 120, or 240 minutes.

The ARVmobile 1.0 for Android was developed in Android 2.2 Froyo [49] with the Eclipse Integrated Development Environment (IDE) 3.7 for Java developers [50] and the Android Development Tool plugin 20.0.2 [51], on MAC OS 10.7 [52], Ubuntu OS 11.10 [53], and Windows OS 7 [54]. The ARVmobile 1.0 for BlackBerry was developed in BlackBerry Java application development 5.0 [55], with the Eclipse IDE 3.6.5 for Java developers [50] and the Blackberry Java plugin 1.5 [56], only on Windows OS 7. Both applications were developed with Java Class Thread [57] as the thread of program execution. The Java Virtual Machine allows an application to have multiple threads of execution running concurrently, so that the smartphone maintains normal operations while receiving real-time BP and HR measurements from the sensor.

### 3.3. Communication between ABPM50 and Smartphone

Wireless communication between the ABPM50 and smartphone is via Bluetooth. The connection is initiated by setting the smartphone on discovery mode, allowing it to detect the ABPM50 and establish Bluetooth pairing [25]. When the devices detect each other, the smartphone prompts the ABPM50 passkey, sends it to the ABPM50, and validates that the same passkey has been sent and received. If the passkey is verified, secure Bluetooth pairing occurs, and data can be exchanged. To save smartphone energy, the smartphone remembers the Bluetooth pairing and is automatically switched to hidden mode after receiving monitoring readings and changed to discovery mode again before the ABPM50 sends new readings.

The smartphone must know the Media Access Control address of the ABPM50 that uniquely identifies each device of a BAN. Radio frequency communication [25] must be used as transport protocol to share the communication channel with other Bluetooth devices. 

### 3.4. Setting Parameters for the ARVmobile 1.0

**Figure 4:**
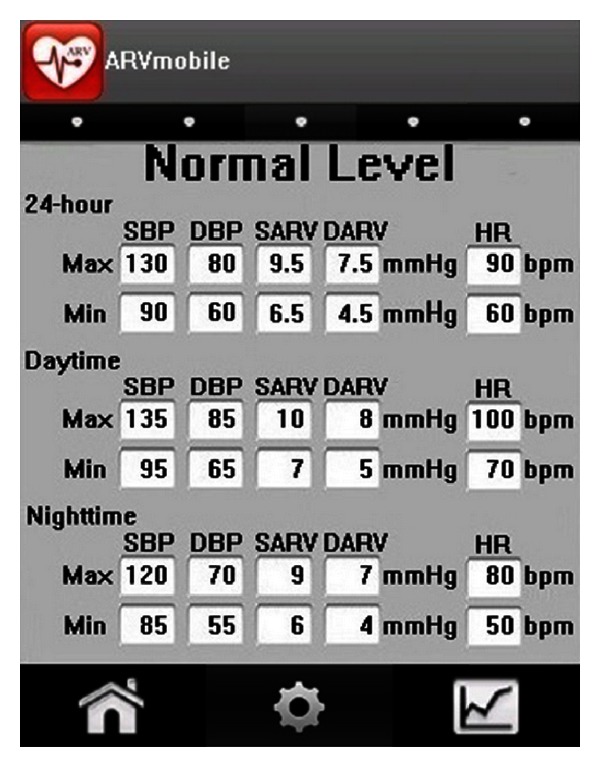
ABP thresholds set on the Blackberry graphic user interface.

**Figure 5:**
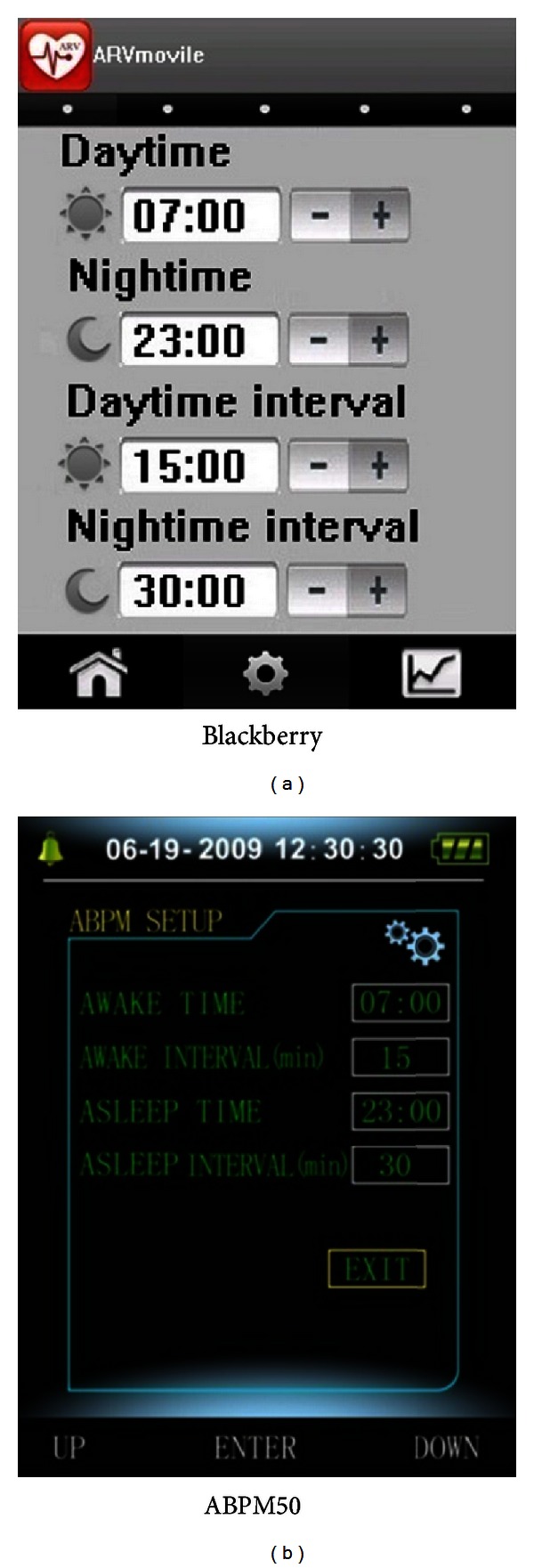
Matching daytime and nighttime thresholds and recording intervals on a Blackberry and ABPM50 graphic user interface.

Before using the ARVmobile 1.0 to monitor ABP and HR, threshold settings for daytime and nighttime periods must be set correspond to the waking and sleeping patterns of the user (Figure 4). The time intervals between two consecutive readings, which can differ for daytime and nighttime periods, must also be set. According to current clinical guidelines [58, 59], readings should be taken at intervals of 30 minutes or less, and the daytime interval must be smaller than or equal to the nighttime interval. In the current, experimental version of the ARVmobile, the threshold settings for daytime and nighttime periods of the ARVmobile and ABPM50 must match (Figure 5), so that the smartphone can determine when to enable (receiving readings) and disable (saving energy) the Bluetooth. Although the ARVmobile was designed to use a sensor without on-board processing and to establish a master-slave relationship controlled by the smartphone, current market restrictions did not allow this type of implementation.

### 3.5. Accuracy of the ARVmobile Monitoring

The goal of the ARVmobile 1.0 and other medical diagnostic devices is to maximize diagnosis accuracy by providing a sufficient amount of accurate data. In ABP monitoring, movement and physical activity often result in invalid ABP readings [60], and recordings can also fail for technical reasons (e.g., improper cuff fitting and auscultatory gap) or patient conditions (e.g., cardiac arrhythmia, rapid pressure changes, severe shock, and HR extremes). A recent review of 25 papers on ABP monitoring found that at least 10% of readings are invalid [61]. Another publication concluded that ABP recordings are successful when at least 85% of readings are suitable for analysis [62]. Current guidelines for management of hypertension indicate that ABP monitoring should be repeated if the recording has fewer than 70% of the expected number of valid values [58]. Thus, there is no specific minimum percentage of valid measurements used to determine the accuracy of ABP monitoring, but there is agreement that a percentage of invalid readings is allowed.

**Figure 6:**
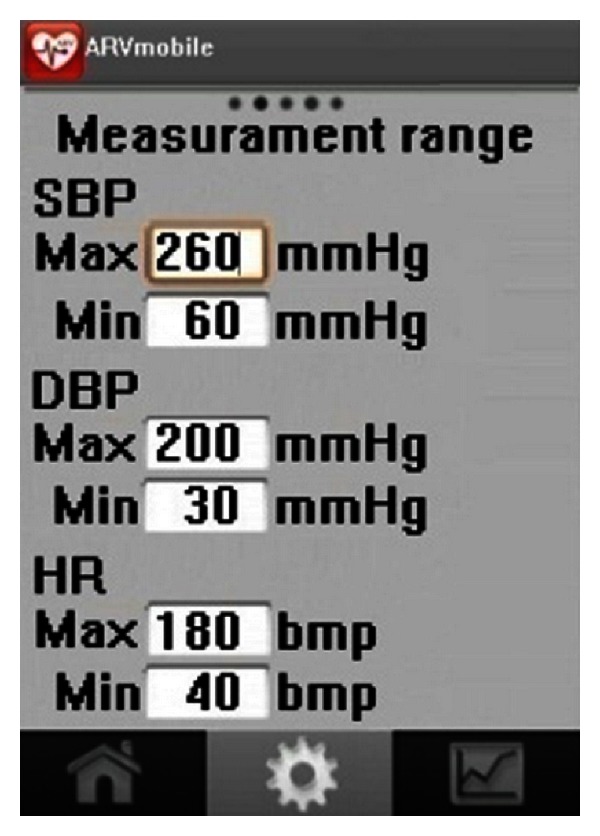
Measurement thresholds set on the Android graphic user interface.

To deal with outlying values, each ABP device has set ranges of BP, ARV, and HR that determine inclusion or exclusion of a reading, which cannot be modified by the user. The ABPM50 has a broader range of operating parameters than other commercial ABPM devices (Table 1), which could increase the number of erroneous readings recorded. Therefore, the measurement thresholds of the ARVmobile 1.0 (Figure 6) were based on those used by the SpaceLabs 90207, which is considered to be an accurate ABP monitor [63–65].

### 3.6. Estimating ARV with the ARVmobile

The ARVmobile 1.0 computes weighted averages of BP and HR, based on the time intervals between consecutive valid measurements, for 24-hour, daytime, and nighttime periods [6]. Weighted averages provide more accurate estimates than standard averages, which assume that all measurements contribute equally [66]. ARV for a specified period is calculated using

(1) $$\begin{array}{c} \text{ARV}=\frac{1}{\sum w_{k}}\sum_{k=1}^{n}w_{k}\times \left|\text{B}{\text{P}}_{k+1}-\text{B}{\text{P}}_{k}\right|, \end{array}$$

where *n* is the number of valid BP readings, *k* ranges from 1 to *n*, and *w*_*k*_ is the time interval between BP_*k*_ and BP_*k*+1_; *w*_*n*_ is the time difference between BP_1_ and BP_*n*_.

**Figure 7:**
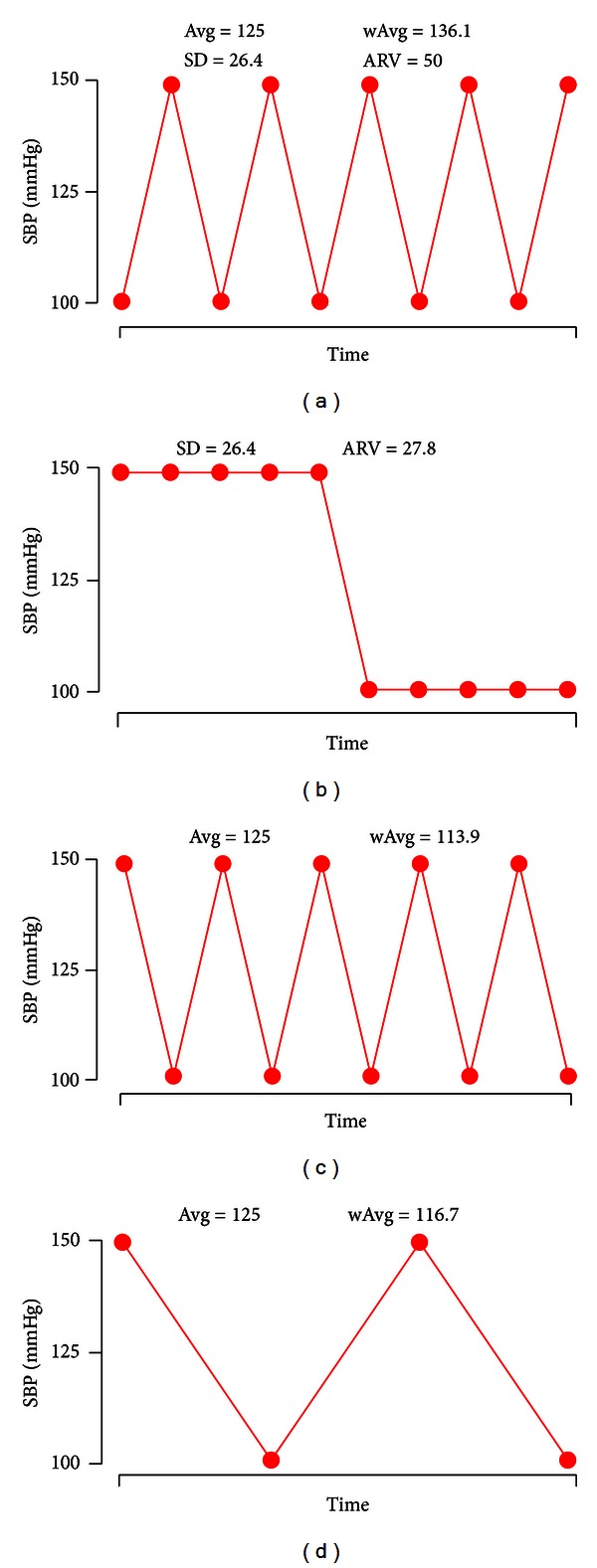
Estimates of average BP (Avg), weighted average BP (wAvg), standard deviation (SD), and average real variability (ARV) for four different systolic blood pressure (SBP) profiles, recorded at intervals of 15 min. The number of values is lower in (d), due to invalid readings that were not recorded. In all four cases, Avg = 125 mmHg.

Using weighted averages and ARV to monitor BPV has several advantages. For example, although the BP recording in Figure 7(a) is visibly more variable than the recording in Figure 7(b), the SD values are the same, because SD reflects only dispersion of values around the mean and not their temporal distribution [67]. In contrast, ARV computed for the more variable recording in Figure 7(a) is almost twice that for the recording in Figure 7(b), providing a more accurate measure of temporal variability in BP.

All of the BP profiles in Figure 7 have a 50% ambulatory SBP load (percentage of systolic readings > 140 mmHg). This ABP parameter improves sensitivity and specificity in the diagnosis of hypertension [68, 69]. Patients with mild hypertension who have an ambulatory SBP load >40% should be strongly considered for antihypertensive therapy [70]. In agreement with this indicator, the weighted average BP (wAvg) for the profile in Figure 7(a) is 136.1 mmHg, which is close to the systolic cutoff for normality in most hypertension guidelines [70], while the standard average BP (Avg) is 125 mmHg, which is between optimal and normal ABP level [71, 72]. Thus, the use of weighted values provides a more accurate hypertension diagnosis.

Figures 7(a) and 7(c) present similar BP profiles, but the record in Figure 7(c) starts with a high SBP and ends with a lower value, similar to a normal circadian, or “dipper,” pattern in which BP decreases during sleep and rises sharply upon awakening [73]. Several studies have confirmed that high nocturnal BP, as seen in Figure 7(a), predicts a higher rate of cardiovascular complications [16–18]. This nondipper status is reflected in the higher wAvg in Figure 7(a), because as recommended, the ABP device is programmed with longer nighttime than daytime intervals, resulting in a higher weighting factors. The dipper pattern is also observed in Figure 7(d), which has a lower frequency of BP recordings. In this case, wAvg corrects for discarded readings by using higher weighting factors, so that wAvg is similar for Figures 7(c) and 7(d). Although Avg for all four profiles is the same, wAvg computed for Figures 7(c) and 7(d) reflect normal ABP levels and are lower than wAvg for Figure 7(a). Thus, wAvg takes into account effects of BP load, noddipper status, and discarded readings.

### 3.7. The ARVmobile Feedback

**Figure 8:**
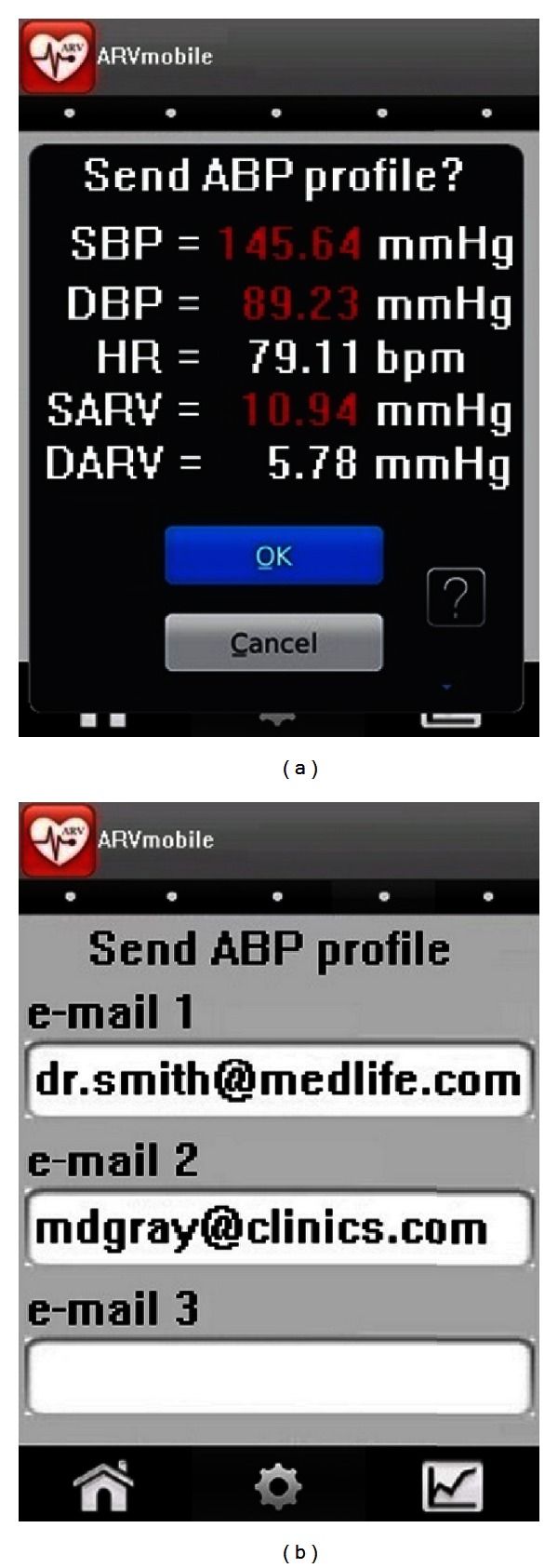
The ARVmobile-Blackberry graphic user interface for (a) instantaneous feedback and (b) forwarding an ABP profile to selected recipients.

**Figure 9:**
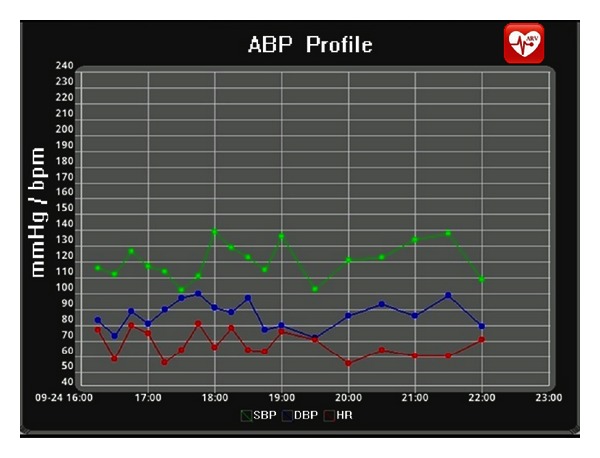
The ARVmobile-Blackberry graphic user interface showing a circadian pattern in ABP.

**Figure 10:**
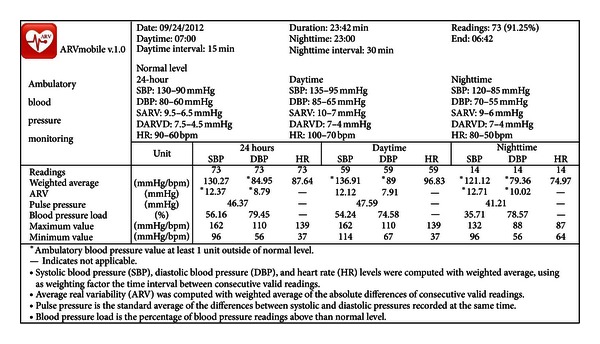
Report forwarded by the ARVmobile to specified healthcare providers.

**Figure 11:**
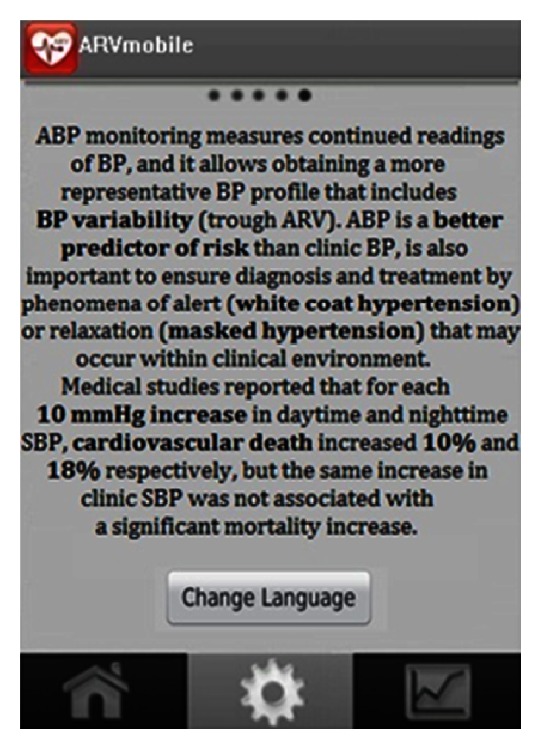
ARVmobile-Android graphic user interface allowing change of language.

The goals of the ARVmobile 1.0 are to identify abnormal levels of traditional or novel cardiovascular risk factors and provide timely feedback to professional healthcare providers. To accomplish these goals, the ARVmobile 1.0 uses a simple GUI (Figure 8) that is user-friendly for older patients, allowing them access to instantaneous feedback and alerting them about abnormal values that are highlighted in red. The ARVmobile 1.0 also provides a graphic ABP profile (Figure 9) that can be scrolled sideways on the screen. The user has the option of sending ABP results via e-mail to specified third parties, such as family members, and sends a complete output report in PDF format to specified healthcare professionals (Figure 10). The report includes 24-hour, daytime, and nighttime BP and HR levels, ARV, BP load, pulse pressure (differences between SBP and DBP [74]), and identification of abnormal APB values outside of the set thresholds. A graphic presentation of the circadian pattern (Figure 9) can be integrated into the PDF file or sent separately in JPGE format. The ARVmobile 1.0 allows feedback to be forwarded to up to three e-mail addresses and can be configured to automatically send ABP and HR profiles at the end of each monitoring period or only after monitoring periods in which abnormal values are detected. The report can be sent in Spanish or English (Figure 11).

### 3.8. The ARVmobile Trial

At the time of writing this paper, over twenty-one subjects aged higher than 51 (mean age 58.9 ± 6.1 years; 61.9% women) have used the ARVmobile 1.0. Volunteer subjects without history of cardiovascular disease and hypertension were recruited in the mobile computer laboratory of the Polytechnic University of Sinaloa, in Mazatlan, Mexico. Recruited participants had reasonable computer skills and were assertive about using new technologies. Informed consent was obtained from every participant. Participants were provided of an ABPM50 device and an Android or Blackberry smartphones and were instructed how to use the PHM application and ABP sensor and to continue with their usual daily activities. The ABPM50 was programmed to obtain readings at 15 minute intervals during awake time (06:00–22:59) and at 30 minutes intervals for the sleeping period (23:00–05:59). The median number of ABP readings was 70 (5th to 95th percentile, 62–79) with a high percentage (>75%) of valid readings. To validate the accuracy of the wireless communication and data processing, BP and HR readings from each subject recorded in the PHM system were compared against those captured by the ABP monitor, without finding inconsistencies between the ABPM50 and ARVmobile records. A posterior survey indicated that the majority of the participants found the ARVmobile 1.0 easy to use and considered the time spent learning how to use the PHM application reasonable. The preliminary results demonstrate that the ARVmobile 1.0 application could be used to perform a detailed profile of ABP and HR in an ordinary daily life environment.

## 4. Discussion

The ARVmobile 1.0 is the result of an interdisciplinary effort in clinical research, data mining analysis, and development of mobile applications. Development focused on (i) an innovative mobile PHM application; (ii) support of early diagnosis and intervention of CVDs, based on cardiovascular risk factors and pattern recognition [75–78] identified by clinical studies [4, 79–82]; (iii) novel strategies to improve adoption by user-friendliness for elderly people.

The inclusion of the capability of calculation of ARV is a major innovation of the ARVmobile 1.0. Although ARV has been shown to be an accurate indicator of BPV [4–6, 9, 10], as well as a significant and independent predictor of cardiovascular complications, when adjusted for BP level and other covariates [4–8], its use to date has been primarily for research, rather than for clinical purposes. However, the clinical use of BPV via calculation of ARV could improve the diagnosis and prognosis of hypertension by providing better information on progressive and end-point organ damage associated with high BP values. Furthermore, BPV could be useful in assessing the efficacy of antihypertensive agents [83] and developing new therapeutic drugs to treat hypertension [84].

The use of weighted averages (wAvg) to construct BP and HR profiles is another major innovation of the ARVmobile 1.0, and wAvg takes the sequential order of measurements into account and, therefore, more accurately represent effects of intermittent stress on the cardiovascular system. Effects of intermittent BP load on cardiovascular structures might be as important as tonic BP load [43], and estimation of ARV and wAvg incorporates this phenomenon.

An important goal of the ARVmobile 1.0 is to provide more effective personal surveillance of elderly hypertensive patients, resulting in a personally tailored approach to hypertension control. One previous study showed that people who check their own BP tend to be more conscious of the importance of taking their medication on a regular basis [85]. The user might notice resistance to antihypertensive drugs or specific foods and activities that raise their BP, and they can adjust their lifestyle, accordingly.

Although use of mobile PHMs could improve and support the lives of the elderly, seniors are often viewed as luddites, reluctant to use modern technology. Thus, development of the ARVmobile 1.0 considered user acceptance and adoption specifically with respect to elderly populations. The first barrier is hardware reluctance. However, a recent market study reported that the 6.2 million working Americans aged 65 and older increased smartphone ownership by 150% during 2007–2010 [86]. The acceptability and ethical aspects of body sensors also could be an important consideration to elderly users. However, widespread use of other personal technologies, such as the Bluetooth headset and smartphone, suggests that older people will easily adjust to wearing sensors for long periods outside of clinical environments [20]. The ARVmobile 1.0 was purposefully designed to be user-friendly to elderly patients with reduced vision and manual dexterity. It has a simplified GUI with a bright screen, large text and numbers, and simple input buttons with touchscreen technology, all of which have been proven to be efficient for older adults [87]. 

The usability of any application depends on the acquisition of new procedural knowledge for proper operation and interaction; therefore, simple design makes the application more usable [88]. This is especially true for older users, because cognitive performance commonly slows down with age. The ARVmobile 1.0 has only three interactive menus (home, settings, and graphical trend). Only the settings menu contains submenus, and these are usually adjusted only once. Security mechanisms, such as user identification for access, were omitted, because users can activate such mechanisms through the security settings of the smartphone. To assure privacy, reports forwarded to selected recipients lack personal identification, which is already associated with the source e-mail address. The ability to operate the ARVmobile 1.0 in either Spanish or English is another user-friendly feature (Figure 11).

Perhaps the most important factor in acceptance and adoption of a mobile PHM system is the user's motivation, which depends on their understanding of the magnitude of the health problem and the benefits of the mobile application. To motivate potential users of the ARVmobile 1.0, it is necessary to disseminate information about the importance of prevention and control of hypertension. Although global prevalence of hypertension is high [2], more than 50% of hypertensive individuals are unaware of their condition [89], and control of hypertension is only around 13.5% [90]. Awareness of isolated systolic hypertension (ISH), which involves high SBP and normal DBP and is more common in the elderly [91], is particularly appropriate to the ARVmobile 1.0. ISH is a major cardiovascular risk factor: each 20/10 mmHg increase doubles the risk for older hypertensive patients [92]. In older patients with ISH, ABP is a better predictor of risk than clinical BP measurements [93, 94]. Cardiovascular death increased 10% and 18%, respectively, for each 10 mm Hg increase in daytime and nighttime SBP, but the same increase in clinical SBP was not associated with a significant mortality increase [93]. The ARVmobile 1.0 presents some of this information in the language change option (Figure 11).

Use of the ARVmobile 1.0 could help avoid overtreatment of elderly patients with white coat hypertension or lack of treatment for those with masked hypertension [95]. Both issues are associated with cost effectiveness, as well as diagnosis and treatment. The cost of hypertension control based on conventional clinical BP measurements can be up to four times higher than control based on ABP monitoring [96], due to unnecessary drug therapy for patients with white coat hypertension [97, 98]; in addition the cost-benefit ratio would be expected to increase, as the cost of managing hypertension rises with increasing rates of diagnosis and prescribing of new, more expensive antihypertensive [62]. Furthermore, the actual raising trend to increase the development of low-cost medical devices [99], and the constant rise of physician fees for primary care [100], could also promote the use of personal ABP monitoring, because in clinical practice, traditional ABP monitors need to be attached and detached by skilled medical technologists [101]. 

The main limitation of usability and adoption of the ARVmobile 1.0 is the ABP sensor design, which must integrate wearability, accurate measurement, power source miniaturization, low power use in reading biosignals and wireless transmission, and secure data transference. To deliver truly personalized health care, the biosensors must be invisible to the user, avoiding activity restriction or behavior modification [20]. Biosensors should be small and lightweight, which depends largely on the size and weight of batteries. However, battery capacity is directly proportional to size [102]. The gold standard for measuring BP with ABP devices is the oscillometric method, which requires inflating a cuff around the arm and requires high power. Development of cuffless sensors that use other BP measurement techniques, such as pulse arrival time [103, 104], but still provide accuracy, is necessary. Another option would be to integrate the sensor into nonclothing items that are already worn by patients. The cost of such novel technology could emerge as the new limitation.

Future development of the ARVmobile requires defining the minimum number of ABP readings required to assess BPV in a reliable or reproducible manner, even with conventional ABP monitors such number is not known [82]. Several studies found that short-term, reading-to-reading BPV estimated by ABP monitoring had poor reproducibility compared to ABP level [105–108], which could account for the rather diverse findings regarding the clinical value of BPV as a predictor of cardiovascular outcomes. The reproducibility of ARV could be improved by increasing the number of BP readings, but 24-hour ABP monitoring can be uncomfortable, especially for elderly patients. Therefore, it is necessary to determine a minimum range of BP readings to calculate ARV without significant loss of information, because accurate calculation of ARV with an adequate number of BP measurements might have great relevance for clinical purposes, and its final implementation in the ARVmobile 1.0 would improve the adoption and use of PHM applications for CVDs prevention. In this sense, the ARVmobile could be a useful and cost-saving PHM system to perform a detailed profile of ABP and HR in an ordinary daily life environment and to estimate potential diagnostic thresholds of abnormal BPV measured as ARV.

## Figures and Tables

**Table 1:** Measurement range of different ABP monitors.

Manufacturer	Model	SBP range	DBP range	HR range
CONTEC MS	ABPM50	10–270	10–270	40–240
SunTech	Oscar 2	25–260	25–260	40–200
Well Alynch	6100S	60–250	25–200	40–200
SpaceLabs	90207	60–260	30–200	40–180
A&D Medical	TM-2430-DP	60–280	40–160	30–200
